# RETRACTION: Evaluating Traditional Chinese Medicine (TCM) Jie Geng and Huang Qi Combination on Reducing Surgical Site Infections in Colorectal Cancer Surgeries: A Systematic Review and Meta‐Analysis

**DOI:** 10.1111/iwj.70621

**Published:** 2025-04-21

## Abstract

**RETRACTION:** ChenS. , TianX. , LiS. , WuZ. , LiY. , LiaoT. , and LiaoZ. , “Evaluating Traditional Chinese Medicine (TCM) Jie Geng and Huang Qi Combination on Reducing Surgical Site Infections in Colorectal Cancer Surgeries: A Systematic Review and Meta‐Analysis,” International Wound Journal 21, no. 2 (2024): e14769, 10.1111/iwj.14769.38351506 PMC10864683

The above article, published online on 13 February 2024, in Wiley Online Library (http://onlinelibrary.wiley.com/), has been retracted by agreement between the journal Editor in Chief, Professor Keith Harding; and John Wiley & Sons Ltd. Following an investigation by the publisher, all parties have concluded that this article was accepted solely on the basis of a compromised peer review process. The editors have therefore decided to retract the article. The authors did not respond to our notice regarding the retraction.

**RETRACTION:** S. Chen , X. Tian , S. Li , Z. Wu , Y. Li , T. Liao , and Z. Liao , “Evaluating Traditional Chinese Medicine (TCM) Jie Geng and Huang Qi Combination on Reducing Surgical Site Infections in Colorectal Cancer Surgeries: A Systematic Review and Meta‐Analysis,” International Wound Journal 21, no. 2 (2024): e14769, 10.1111/iwj.14769.38351506 PMC10864683

The above article, published online on 13 February 2024, in Wiley Online Library (http://onlinelibrary.wiley.com/), has been retracted by agreement between the journal Editor in Chief, Professor Keith Harding; and John Wiley & Sons Ltd. Following an investigation by the publisher, all parties have concluded that this article was accepted solely on the basis of a compromised peer review process. The editors have therefore decided to retract the article. The authors did not respond to our notice regarding the retraction.

